# Reflex microsatellite instability & immunohistochemistry testing practices & follow-up of abnormal results at U.S. cancer programs

**DOI:** 10.1186/1897-4287-9-S1-P2

**Published:** 2011-03-10

**Authors:** Laura C Beamer, Marcia Grant, Deborah J MacDonald, Heather Hampel, Kathleen Blazer, Carin Huizenga, Jeffrey N Weitzel

**Affiliations:** 1 Division of Clinical Cancer Genetics, City of Hope National Medical Center, Duarte, CA 91010, USA; 2 Division of Nursing Research & Education, City of Hope National Medical Center, Duarte, CA 91010, USA; 3 Division of Human Genetics, Ohio State University, Columbus, OH 43240, USA

## Background

Abnormal results of microsatellite instability (MSI) or immunohistochemistry (IHC) testing of colorectal (CRC) tumors may suggest the presence of Lynch Syndrome (LS), a hereditary cause of CRC. Individuals with LS are at high risk for second primary cancers. Abnormal results of MSI and IHC testing influence care decisions. MSI is also a prognostic marker. The purpose of this survey-based study is to determine the current MSI and IHC testing practices and subsequent follow-up of abnormal results in U.S. cancer programs. The study aims include to: 1) explore the practice of MSI/IHC testing based on type of cancer program and the number of CRC cases accessioned during 2009 and 2) identifying the process and factors associated with follow-up of abnormal results. Routine MSI and IHC testing of CRC tumors is an emerging practice in the U.S. To date, no published studies were found that describe the practice of reflex testing for MSI and IHC abnormalities on colorectal tumors in the U.S.

## Methods and materials

A study packet (i.e., letter of invitation, survey, $5.00 incentive, and postage-paid return envelope) will be mailed to the cancer program and tumor registry directors at the 40 NCI-designated Comprehensive Cancer Centers, 567 ACS-accredited Community Hospital Comprehensive Cancer Programs, and 521 ACS-accredited Community Hospital Cancer Programs in the U.S. Collaboration between the directors of the cancer program and tumor registry will be encouraged and only one set of survey responses per cancer program will be retained. The survey was developed based on input from an internal and external panel of experts and revised following pilot testing in 22 centers (99.5% return rate). The survey includes 11 check-box items of which 6 also have an option to write in comments. Recruitment will follow Dillman's method (i.e. pre-notice letter; study packet, thank you/reminder postcard; repeating up to 2 cycles). Center characteristics and MSI/IHC practices will be summarized using descriptive statistics. Chi square tests will explore associations between type of cancer program and MSI/IHC testing practices. Analysis of variance or planned comparisons will examine differences between program’s CRC cases in 2009 and the type of cancer program. Content analysis will be used to analyze narrative responses to survey questions.

## Conclusions

The findings of this study may influence (and ultimately enhance) uptake and translation of MSI and IHC testing practices for newly diagnosed CRC tumors, which may lead to improved cancer screening and prevention practices.

